# The Role of Regulatory B Lymphocytes in Allergic Diseases

**DOI:** 10.3390/biomedicines12122721

**Published:** 2024-11-27

**Authors:** Agnieszka Lipińska-Opałka, Michalina Leszczyńska-Pilich, Agata Będzichowska, Agata Tomaszewska, Agnieszka Rustecka, Bolesław Kalicki

**Affiliations:** 1 Faculty of Medicine, University of Warsaw, 02-089 Warsaw, Poland; awawrzyniak@wim.mil.pl (A.T.); kalicki@wim.mil.pl (B.K.); 2 Department of Pediatrics, Nephrology and Allergology, Military Institute of Medicine–National Research Institute, 01-141 Warsaw, Poland; mleszczynska@wim.mil.pl (M.L.-P.); abedzichowska@wim.mil.pl (A.B.); arustecka@wim.mil.pl (A.R.)

**Keywords:** regulatory B cells, immune system, asthma, atopic dermatitis, allergic rhinitis, food allergy, allergen immunotherapy

## Abstract

Purpose of review: Regulatory B cells (Bregs) are a key component in the regulation of the immune system. Their immunosuppressive function, which includes limiting the inflammatory cascade, occurs through interactions with other immune cells and the secretion of cytokines, primarily IL-10. As knowledge about B cells continues to expand, their diversity is becoming more recognized, with many subpopulations identified in both human and animal models. However, identifying specific transcription factors or markers that could definitively distinguish regulatory B cells remains a challenge. This review summarizes recent findings on the role of B regulatory cells in allergic diseases. Recent findings: In patients with bronchial asthma, atopic dermatitis, and food allergies, the number of regulatory B cells is reduced, and disease severity is inversely proportional to the quantity of these cells. Furthermore, in patients with atopic dermatitis, the ability of regulatory B cells to produce IL-10 in response to IL-6 stimulation is diminished. However, allergen immunotherapy has been shown to induce the formation of regulatory T cells as well as regulatory B cells. Summary: The success of future therapies based on B cells may depend on deepening our current understanding of their phenotypes, induction, differentiation, and function. Research in these areas is essential for understanding the mechanisms regulating Breg activity and for developing potential targeted therapies in the treatment of allergic diseases.

## 1. Introduction

The immune system plays a crucial role in defending the body against pathogens, but when it malfunctions, it can also contribute to the development of certain diseases. B lymphocytes, as an integral part of the adaptive immune system, are responsible for various functions, including antigen presentation and antibody production. A particularly significant subgroup is regulatory B lymphocytes (Bregs), which have the ability to suppress immune responses and regulate inflammatory processes in the body [1].

B lymphocytes originate in the bone marrow and undergo differentiation and maturation in peripheral lymphoid organs such as lymph nodes and the spleen. Their key role is not only to mount responses against pathogens but also to introduce regulatory mechanisms that can prevent excessive immune reactivity [2].

Bregs, which make up about 0.5% of all B lymphocytes, play an important role in controlling the inflammatory cascade [3]. Their function becomes particularly relevant in the context of allergic diseases, where an excessive immune response to harmless antigens can lead to inflammatory states causing various clinical symptoms. Dysfunction of regulatory B lymphocytes, whether in the form of deficiency or overactivity, is also associated with other conditions, including autoimmune diseases, cancers, and transplant rejection [4].

Previous research on Breg function has focused on their differentiation, maturation, and immunomodulatory properties, particularly the cytokines they secrete, which affect the function of other immune cells [5]. Understanding the role of regulatory B lymphocytes in the context of allergic diseases opens new avenues for the treatment and prevention of these conditions.

**Figure 1:**
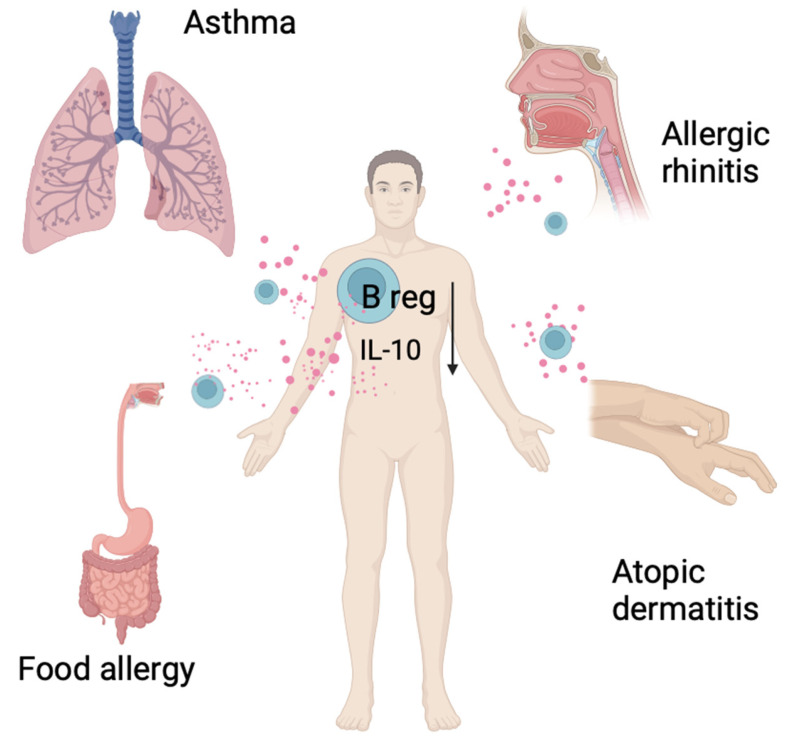
Allergic diseases associated with reduced numbers of regulatory B lymphocytes and their cytokines, mainly IL-10.

Research on Bregs and their mechanisms of action may contribute to the development of new therapeutic strategies that are personalized and effective in managing autoimmune diseases and allergies. The aim of this review was to place the role of regulatory B cells in the pathogenesis of selected allergic diseases such: asthma, atopic dermatitis, allergic rhinitis, food allergy and to discuss their role in the only causal therapy of allergy, which is allergen immunotherapy. The literature considered in this review exclusively pertains to studies conducted on humans models (Figure 1).

## 2. B-Cell Development, Activation and Differentiation

Lymphocytes B can be categorized by their functions and developmental stages. The differentiation and maturation of B lymphocytes is a multi-stage and complex process. The various stages of B cell differentiation and maturation can be grouped based on the expression of surface and intracellular markers, cell cycle profiles, and the status of light and heavy chain gene rearrangements [6].

In mammals, the early stages of B cell development occur in the fetal liver, followed by the bone marrow [7]. The progenitor pre-pro-B cell located in the bone marrow contains germline immunoglobulin genes and expresses the markers B220 and CD34 [8]. As B cells progress to the pro-B stage, they lose the CD34 marker and transition into pre-B cells in humans [9]. In mice, pro-B cells lose the CD43 marker and become pre-B cells [10].

A key characteristic of pre-B cells is the presence of immunoglobulin heavy chain μ (mi(u)) protein in their cytoplasm, along with the pre-B cell receptor (pre-BCR). The pre-BCR is composed of proteins known as surrogate light chains (SLC), which consist of the mi(u) protein coupled with two other proteins. The presence of the pre-BCR on the surface of B cells allows signaling into the cell, indicating proper heavy chain assembly [11].

Additionally, the pre-BCR facilitates the recombination of the kappa (κ) light chain. Only cells with a fully formed receptor receive signals for further proliferation. Subsequently, pre-B cells transition into immature B cells in both humans and mice. Immature B cells are characterized by the expression of IgM and the presence of the BCR on their surface. To prevent the formation of autoreactive B cells, transitional B cells undergo processes of deletion, receptor editing, and anergy [6,10,12].

Approximately 3% of immature B cells develop into mature B cells. Most immature B cells leave the bone marrow in both humans and mice as transitional B cells, expressing the CD24 marker [13]. Transitional B cells in mice are classified into three known subtypes: T1, T2, and T3. This classification has not yet been identified in humans. Transitional B cells then differentiate into naive mature B cells located in the spleen, which can further differentiate into marginal zone B cells, follicular B cells, or plasmablasts [6].

Naive B cells express BCRs of the immunoglobulin M (IgM) and D (IgD) classes on their membrane [14]. Antigen activation of BCRs leads to their activation and proliferation. The next stage involves the formation of a germinal center (GC), which is a site for B cell proliferation. Plasma cells, which are effector cells derived from plasmablasts, function to produce immune antibodies. Plasmablasts can arise from naive mature B cells, marginal zone B cells, or follicular B cells. B cells that produce low-affinity antibodies or autoreactive antibodies (autoantibodies) are eliminated. B cells with high-affinity antibodies differentiate into plasma cells or memory cells. Additionally, pre-GC B cells located in the spleen, expressing CD19, CD38, and IgD, can form germinal centers. Germinal centers can differentiate into both plasmablasts and memory cells. Memory cells are characterized by the expression of CD19 and CD27 markers and have the ability to transform into plasmablasts, which then differentiate into effector cells [6,15].

## 3. Regulatory B Cells

In the 1970s, the immunoregulatory function of B cells was first described. Studies conducted in a delayed hypersensitivity model in guinea pigs, using cyclophosphamide and transfer of splenocytes depleted of B cells, demonstrated that animals with a reduced ratio of B cells to T cells exhibited a more intense and prolonged hypersensitivity reaction compared to the control group. These findings suggested a potential suppressive function of B cells in relation to T cells [16].

In the 1990s, an experimental model of experimental autoimmune encephalomyelitis (EAE) was developed in mice, in which CD4+ T cells play a critical role. Additionally, some of these mice were depleted of B cells. While the severity of EAE was comparable between the experimental and control groups, the B cell-deficient mice exhibited a prolonged disease duration [17].

At the beginning of the 21st century, research demonstrated that B cells could induce immunosuppressive mechanisms through IL-10 in mouse models of EAE, chronic colitis. It was shown that IL-10 functions as a regulator of the immune system [18].

The first studies suggesting an immunoregulatory effect of B cells in humans came from observations of patients treated with rituximab–a humanized anti-CD20 monoclonal antibody that depletes B cells, used e.g., in kidney transplant recipients to prevent rejection. The majority of patients treated with rituximab developed acute rejection mediated by T cells [19], and some of these patients also experienced the onset of psoriasis [20] and exacerbation of ulcerative colitis symptoms [21,22].

Over the past 30 years of research on regulatory B cells, several phenotypically diverse groups of regulatory B cells, referred to as Bregs, have been identified. These cells are transiently or persistently present in various organs and at different stages of B cell differentiation. Their full role remains unknown, both in humans and animals [23].

The immunosuppressive function of Bregs is primarily based on the secretion of immunosuppressive cytokines, with IL-10 initially considered the main mediator. However, new studies indicate that IL-35 plays an equally significant role [24]. Both cytokines inhibit CD4+ T cell proliferation, reducing the inflammatory Th1 response and promoting the expansion of regulatory T cells (Tregs) [25].

Other substances secreted by Bregs include transforming growth factor-β (TGF-β), which promotes Treg production, and granzyme B, which inhibits T cell activity [26,27]. Bregs also exert immunosuppressive effects through interactions with surface ligands, such as programmed death-ligand 1 (PD-L1), which binds to T cells via the PD-1 receptor [28]. Additionally, Bregs can induce apoptosis through tumor necrosis factor-related apoptosis-inducing ligand (FasL) binding to the Fas receptor on CD4+ and CD8+ T cells [29].

Recent studies have pointed to alternative mechanisms of immunosuppression by Bregs, both in humans and animal models, involving CD39, CD73, the aryl hydrocarbon receptor (AhR), and others [1,2,30].

The phenotypic diversity of Bregs suggests that B cells at various developmental stages may acquire immunoregulatory properties upon stimulation by various factors. To date, no single specific transcription factor has been identified as responsible for this transformation. IL-10, IL-21, B cell receptors (BCR), PMA activators, Toll-like receptor ligands, and intracellular signaling molecules such as STAT3 and MyD88 are among the confirmed factors involved in Breg induction [4,28,30,31,32].

Currently, there is no definitive definition or classification of Bregs. Research conducted on both mouse and human models has led to the identification of several Breg subsets with similar phenotypic and functional properties. Below are the Breg subsets identified in humans [15] (Table 1).

## 4. The Role of Regulatory B Cells in Allergic Diseases in Humans

An allergic reaction is a multistep immune response triggered by contact with an allergen. During subsequent exposure to the allergen, an inflammatory reaction develops, characterized by a type 2 immune response involving both the innate and adaptive immune systems. Allergic inflammation can be divided into two phases: the sensitization phase and the effector phase.

The sensitization phase begins with the capture of the allergen by dendritic cells located in the respiratory tract, skin, or gastrointestinal tract. Dendritic cells present the allergen on their surface via MHC class II molecules to naïve CD4+ T cells. Allergen-specific CD4+ Th2 cells, through the secretion of cytokines IL-4 and IL-13, promote class switching to IgE in B lymphocytes. Subsequently, memory B cells are formed, which differentiate into plasma cells that produce IgE, binding to receptors on mast cells and basophils. Upon re-exposure to the allergen, specific Th2 cells and memory B cells undergo rapid proliferation, leading to increased antibody production. The sensitization phase is asymptomatic.

The effector phase is divided into early and late responses. The early response occurs within seconds of allergen exposure and represents a type I hypersensitivity reaction. The binding of the allergen to IgE on the surface of mast cells and basophils triggers their activation and the release of inflammatory mediators such as histamine, proteases, leukotrienes, heparin, lipid-derived mediators, and cytokines, which lead to allergic symptoms. The late response develops several hours after allergen exposure and depends on the target organ, involving T cell or mast cell activation. In some cases, such as in individuals with atopy and asthma, the late-phase response may occur without the involvement of mast cells, instead relying on MHC molecules rather than IgE. This suggests that airway narrowing in asthma patients may result solely from T cell activation [75].

**Figure 2:**
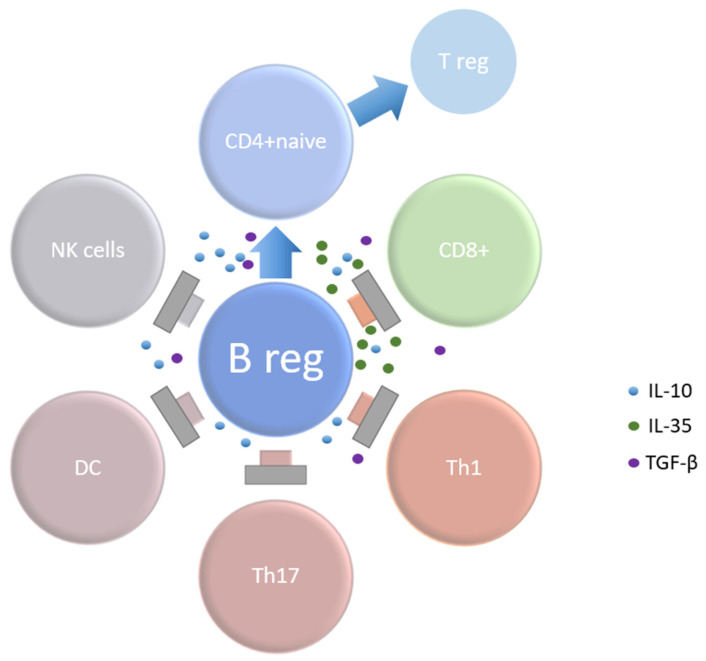
Role of regulatory B cells in the pathophysiology allergic diseases in humans. Bregs, through IL-10, inhibit the Th1, Th17 and CD8+ T cell response, convert naïve CD4+ T cells into regulatory T cell populations and inhibit pro-inflammatory cells such as NK cells (natural killer cells) and DC (dendritic cells). Bregs secrete TGF-β+ which also stimulate naïve CD4+ T cells, generating FoxP3+ Tregs and inducing anergy in CD4+ and CD8+ T cells. Another cytokine produced by Bregs, IL-35+, also promotes the development of Tregs. Abbreviations: Breg = regulatory B cell, DC = dendritic cell, IL = interleukin, NK cell = natural killer cell, DC = dendritic cell, TGF = transforming growth factor, Th = T helper cell, Treg = regulatory T cell.

The role of regulatory B lymphocytes in allergic diseases is not yet fully understood. However, it has been shown that the number of regulatory B cells in the serum of patients with allergic diseases differs from that of healthy individuals. The following section provides a review of the available literature on the presence and activity of regulatory B lymphocytes in various allergic diseases in humans (Figure 2).

## 5. Asthma

Bronchial asthma is an inflammatory disease of the airways and is the most common chronic condition in childhood. It is characterized by the presence of a chronic inflammatory state in the airways, accompanied by hyperreactivity and obstruction. Various endotypes of asthma are recognized, including eosinophilic and neutrophilic asthma [1,15,76].

In eosinophilic asthma, T-helper type 2 lymphocytes are involved in the response to allergens. The function of regulatory B cells is impaired, exhibiting dysfunction manifesting as reduced production of IL-10 and difficulty in stimulating regulatory T cells. IL-10 inhibits IgE-dependent allergic reactions by reducing inflammation in the airways [15,77,78].

The biologic therapies approved for use in patients with eosinophilic asthma include omalizumab, a monoclonal antibody binding to IgE, and dupilumab, a monoclonal antibody targeting the IL-4 receptor [1,15].

It has been shown that in patients with severe asthma, dupilumab treatment results in an increase in the concentration of regulatory B lymphocytes [79]. Qian et al. demonstrated that asthma patients have elevated levels of IL-10 and reduced levels of BCL-3 protein. This indicates that BCL-3 is a negative regulator of IL-10, representing a potential therapeutic target in allergic asthma [80].

Research has indicated that patients with a history of asthma have a lower absolute count and percentage of CD19+CD24hiCD27+ regulatory B cells compared to a control group [81]. Additionally, this type of regulatory B cell produces less IL-10 upon LPS stimulation [77]. In contrast, CpG stimulation does not decrease their production of IL-10 [82]. CD9+ regulatory B cells secreting IL-10 have been identified in both mice and humans, confirming their role in inducing apoptosis in CD3+CD4+CD25+ T cells [43]. Earlier studies have shown that the number of CD9+ Bregs is diminished in mice with a history of asthma [83]. Furthermore, CD9+ B lymphocytes participate in reducing Th2- and Th17-dependent inflammation, increasing the ratio of regulatory to effector T lymphocytes [84]. RSV infection in young infants is a risk factor for future asthma development. Scientific reports indicate that the number of RSV-infected regulatory B cells in newborns corresponds to a decreased number of Th1 lymphocytes [85]. Recent studies have shown that pediatric asthma patients possess lower levels of CD24+CD38+ regulatory B cells producing IL-10 compared to a control group, highlighting the significant role of an appropriate quantity and function of regulatory B cells in controlling allergic inflammation [86].

## 6. Atopic Dermatitis

Atopic dermatitis (AD) is a chronic condition with unclear etiopathogenesis, characterized by skin lesions exhibiting erythema, papules, vesicles, and peeling. Approximately 3% of adults and 20% of children worldwide suffer from this condition [87]. The foundation of treatment includes emollients and topical anti-inflammatory medications, with biologic drugs such as dupilumab applied in severe cases.

Research studies specifically examining the role of Bregs in atopic dermatitis in humans are limited. Some reports indicate a correlation between AD and food allergies, as well as an imbalance between Th1 and Th2 lymphocyte populations [75]. Furthermore, it has been demonstrated that patients with severe AD exhibit a reduced number of CD24hiCD38hi regulatory B cells, with their quantity inversely proportional to the severity of the disease. Additionally, the ability of regulatory B cells in these patients to produce IL-10 in response to IL-6 stimulation is diminished [88].

Eosinophils also play an important role in the pathogenesis of AD. Lee et al. showed that Breg cells, through secretion of IL-10, inhibit eosinophil infiltration into the skin, but also inhibit their activation, including degranulation. Ultimately, this leads to amelioration of AD symptoms [89].

A recent immunophenotypic study of B lymphocytes in AD patients undergoing treatment with dupilumab compared to those not receiving therapy revealed that treated patients had a higher expression of CD200 on their B lymphocytes than the control group [90].

## 7. Allergic Rhinitis

Allergic rhinitis (AR) is a syndrome characterized by symptoms such as itching, paroxysmal sneezing, watery nasal discharge, and nasal mucosal congestion. These are triggered by an IgE-dependent type 1 hypersensitivity reaction to airborne allergens such as dust mites, animal dander, or grass and tree pollen. Allergens can induce an allergic response through various mechanisms [91].

Scientific studies on regulatory B cells (Bregs) have shown a decreased number of CD19+CD24hiCD38hi, CD19+CD25+CD71+CD73-, and CD19+CD25hiCD71+CD73- cells in patients with AR compared to the control group. Additionally, a lower count of CD4+PD-1+CXCR5+ follicular helper T cells (Tfh) was observed. Conversely, the number of CD19+CD24hiCD27+ Bregs, memory B cells, and plasma cells was increased [92]. Another study on IL-10-producing CD19+CD25+CD71+ regulatory B cells found that their number was reduced in AR patients [82]. These observations suggest a potential imbalance in immune regulation, with a reduction in specific Breg populations that may impair tolerance mechanisms, while an increase in memory B cells and plasma cells could reflect heightened immune activation or inflammation.

There are reports of decreased expression of CD25 and the IL-2 receptor alpha chain on regulatory B cells in AR. The IL-2 receptor alpha chain plays a crucial role in the induction of regulatory T cells. Moreover, patients with both AR and asthma show a lower number of Bregs compared to those with AR alone. This suggests that Bregs may play a role in the development of asthma in patients with a history of AR [93].

Another study found that peripheral blood of AR patients contained higher percentages of memory B cells, plasma cells, and CD19+CD24hiCD27+ regulatory B cells (Bregs) than those of healthy controls, while the percentages of naïve B cells and CD19+CD24hiCD38hi Bregs were significantly lower in AR patients than in healthy individuals. These results emphasize the fact that different B cell subpopulations play distinct roles in the pathogenesis of this allergic disease [94].

## 8. Food Allergy

Food allergy is an undesirable immune response to food, classified into IgE-dependent, IgE-independent, and mixed types. It affects approximately 8% of the pediatric population in Western countries. IgE-mediated food allergies are most common in infancy and early childhood. Common allergens during this period include cow’s milk, eggs, peanuts, and tree nuts. While allergies to cow’s milk and eggs often resolve with age, nut allergies tend to persist. IgE-mediated food allergy is based on type I hypersensitivity mechanisms and can lead to anaphylactic reactions. Non-IgE-mediated reactions primarily involve gastrointestinal symptoms and have a delayed onset [95,96,97].

Scientific reports indicate that in cases of cow’s milk allergy, the number of CD19+CD5+ regulatory B cells producing IL-10 and TGF-β is reduced compared to individuals tolerant to milk [98,99,100].

Food allergy and atopic dermatitis (AD) are interrelated. AD is a TH2-dependent disease, independent of IgE [99].

CD5+ IL-10-secreting regulatory B cells and TGF-β-secreting Bregs have been described in IgE-independent food allergy and AD [100]

Additionally, food allergies are a predisposing factor for the development of inflammatory bowel diseases, such as Crohn’s disease and ulcerative colitis [101].

In patients with food allergies and ulcerative colitis, CD19+CD25+CD71+CD73- regulatory B cells produce less IL-10 compared to the control group. Additionally, an increased number of CD4+CD25- T cells was observed in the study group [102].

These findings suggest that regulatory B cells, particularly those producing IL-10 and TGF-β, play a crucial role in maintaining immune tolerance in food allergies and related conditions. Targeting these B cells or their cytokine production may offer promising therapeutic strategies for managing food allergies.

## 9. Allergen Immunotherapy

Allergen immunotherapy (AIT) is a form of etiological treatment aimed at reducing IgE-mediated hypersensitivity to inhaled, food, or insect venom allergens. Allergen immunotherapy involves the repeated administration of increasing amounts of allergen extract or products over several years to induce tolerance. The underlying mechanisms of specific immunotherapy include a reduction in allergen-specific T-helper 2 (TH2) cells, production of IgG and IgA antibodies, and the induction of regulatory B and T cells [103].

An important area of current research is the role of IgG4 antibodies, which are considered crucial for immunological tolerance due to their anti-inflammatory effects [104].

It has been demonstrated that AIT with house dust mite allergens induces a long-term increase in IL-10+ and/or IL-1RA+ regulatory B cells and an increase in IgG4 levels in humans [105].

Studies confirm that regulatory B cells can produce IgG4. A 10- to 100-fold increase in IgG1 and IgG4 immunoglobulins was observed following allergen immunotherapy [49].

Research confirms that allergen immunotherapy in patients with AR symptoms induces IL-35- and IL-10-producing regulatory B and T cells [106]. During pollen season, the number of IL-10-producing regulatory B cells increases in patients allergic to inhaled allergen [95].

Moreover, studies report the role of epigenetic changes in histone acetylation/deacetylation in regulating IL-10 expression [107].

A commonly used model in regulatory B cell research involves beekeepers and venom-specific immunotherapy [30].

Beekeepers are frequently stung by insects during the spring-summer season, with significantly fewer stings in the fall-winter period. Analysis of venom allergen-specific B and T cells in and out of season provides valuable insights into allergen tolerance [75].

In both beekeepers and patients undergoing venom AIT, an increase in the number of CD73-CD25+CD71+IL-10+ BR1 regulatory B cells has been observed [108]. Additionally, it was found that the proportion of IL-10-specific regulatory B cells in response to bee venom allergens is higher compared to non-specific B cells. In contrast, naïve CD27-type Bregs selectively produce more IgG4 than IL-10-non-producing B cells [49].

All these studies highlight the role of Bregs in the development of tolerance to allergens through increased production of IL-10 as well as IgG4 antibodies.

Table 2 presents a summary of the most important studies on regulatory B lymphocytes in allergic diseases.

## 10. Summary

A healthy immune system is characterized by a balance between pro-inflammatory and anti-inflammatory factors. Numerous scientific studies in recent years indicate a significant role of regulatory B cells (Bregs) in the regulation of inflammation. Unfortunately, no specific transcription factors or markers identifying regulatory B cells have been established so far. Understanding the physiology of Bregs is complicated by the fact that their mechanisms are similar to those used by other immune cells, with which they often interact. Breg dysfunction affects the development of allergic diseases in both animal models and humans, including bronchial asthma, atopic dermatitis, allergic rhinitis, and food allergies. Despite strong evidence for their role in reducing inflammation, inducing and maintaining tolerance to allergens, and controlling allergic diseases, research into modifying Bregs in these conditions is still lacking. Further studies are essential to investigate the phenotypes, induction, differentiation, and function of regulatory B cells. Understanding these mechanisms could lead to the development of Breg-based therapies for allergies and other conditions, or the use of Bregs as prognostic markers for the course of specific diseases.

## Figures and Tables

**Table 1:** Types of regulatory B lymphocytes and their characteristics.

A Type of Regulatory B Cell	Location	Phenotype	Immunosuppressive Molecules	Author/Year	Mouse Equivalent
Immature transitional B cell	Peripheral blood, liver	CD19+CD24hi CD38hi CD1dhi	IL-10, CD80/86	Blair et al., 2010, Bosma et al., 2012, Das et al., 2012, Flores-Borja et al., 2013, Liu et al., 2016, Menon et al., 2016, Oleinika et al., 2018 [33,34,35,36,37,38,39]	No
PD-L1 hi B cells	Solid tumors, spleen	CD19+PD-L1hi	PD-L1, IgA, IL-10	Khan et al., 2015, Feres et al., 2019, Sun et al., 2019 [40,41,42]	Yes
CD9+ B cells	Peripheral blood, spleen	CD19+CD9+	IL-10	Brosseau et al., 2018 [43]	Yes
Plasmablasts	Peripheral blood, lymph nodes, spleen	CD19+CD24hi CD27int CD38+CD138+IgA+PD-L1− IL-10+	IL-10, TGF-β	Matsumoto et al., 2014, de Masson et al., 2015, Shalapour et al., 2015, Mao et al., 2017, Fillatreau et al., 2018, Fehres et al., 2019 [41,44,45,46,47,48]	Yes
Br1 cells	Peripheral blood	CD19+CD25+CD71hi CD73lo	IL-10, IgG4	Van de Veen et al., 2013 [49]	No
Tim1+ B cells	Peripheral blood, spleen	CD19+Tim-1+	IL-10	Ding et al., 2011, Xiao et al., 2012, Xiao et al., 2015, Aravena et al., 2017, Gu et al., 2017, Xiao et al., 2020 [50,51,52,53,54,55]	Yes
B10 B cells	Peripheral blood, spleen, astric mucosa, stomach cancer	CD19+CD24hi CD27+	IL-10	Iwata et al., 2011, Yanaba et al., 2008, Sheng et al., 2015, Chien et al., 2017, Murakami et al., 2019, Daien et al., 2021, Meng et al., 2018, Piper et al., 2019 [56,57,58,59,60,61,62,63]	Yes
Granzyme B+ lymphocytes (GraB cells)	Peripheral blood, solid tumors	CD19+CD20+GrB+CD86+CD147+, IDO+, (CD38±CD25±CD27+CD1d±CD5±CD10+IgM±)	Granzyme B, IDO, CD25	Hagn et al., 2009, Lindner et al., 2013, Jahrsdorfer et al., 2006, Chesneau et al., 2015, Kaltenmeier et al., 2015 [26,64,65,66,67]	No
CD5+ B cells	Peripheral blood	CD19+CD5+GrB+ CD1dhi	Granzyme B, IL-10	Hagn et al., 2010, Zhang et al., 2012, Zhang et al., 2014 [68,69,70]	Yes
Adipose tissue B lymphocytes	Adipose tissue	CD19+ CD27+CD38hi	IL-10	Nishimura et al., 2013, Garcia-Hernandez et al., 2018 [71,72]	Yes
CD39+CD73+ B cells	Peripheral blood, spleen	CD19+CD39+ CD73+	AMP/Adenosine	Saze 2013, Kaku 2014 [73,74]	Yes

Abbreviations used: AMP—adenosine monophosphate, Br1—regulatory B lymphocyte type 1, CD—cluster of diferrentation antigen, GrB—granzyme B, IDO—indoleamine 2,3-dioxygenase, Ig—immunoglobulin, IL—interleukin, PD-L1—programmed death-ligand 1, TGF—transforming growth factor.

**Table 2:** Summary of the most important studies on regulatory B cells in allergic diseases.

Disease or Therapy	Feature of Regulatory B Cells	Author/Year
Asthma	Reduced ability of CD24++CD27+ regulatory B cells to produce IL-10 in response to LPS94 stimulation	Van der Vlugt et al., 2014 [77]
	Reduced ability of B regs to produce IL-10 in response to CpG95 stimulation	Wirz et al., 2019 [82]
	Reduced number of CD5+ and CD1d+CD5+ B lymphocytes	Wiest et al., 2019 [109]
	CD9+ regulatory B lymphocytes induce apoptosis of CD3+CD4+CD25+ effector T lymphocytes	Braza et al., 2014, Brosseau et al., 2018 [43,83]
	The number of regulatory B cells infected with RSV is proportional to the viremia and to the reduced number of Th1 lymphocytes in the serum	Zhiyaki et al., 2017 [85]
	Reduced number of B regs in children and adolescents with bronchial asthma	Sheehan et al., 2023 [86]
	Lower absolute number and percentage of B regs in asthma patients compared to the healthy group	Miyaijma et al., 2020 [81]
Atopic dermatitis	The occurrence of regulatory B cells of the CD24hiCD38hi type is reduced, and the severity of the disease is inversely proportional to the number of this type of regulatory B cells. The ability of regulatory B cells to produce IL-10 is lower in response to IL-6 stimulation	Yoshihara et al., 2019 [88]
	B regs, through the secretion of IL-10, inhibit eosinophil activation, including degranulation and EPO secretion.	Lee et al., 2024 [89]
Allergic rhinitis	Increased number of regulatory B lymphocytes of the CD19+CD24hi CD27+ type, decreased number of CD19+CD24hi CD38hi, CD19+CD25+CD71+CD73 and CD19+CD5hiCD1d+ lymphocytes	Kim et al., 2016, Luo et al., 2018 [92,93]
	The occurrence of CD19+CD25+CD71+ regulatory B cells producing IL-10 after TLR9 stimulation is reduced	Wirz et al., 2019 [82]
	Lower percentage of IL-10 secreting Bregs of the CD19+CD24hiCD38hi and CD19+CD5hiCD1d+ type in patients with seasonal ANN compared to the control group. Increased concentration of both above-mentioned Bregs types in patients after treatment with allergen immunotherapy (SCIT, subcutaneous immunotherapy)	Shamji 2019 [95]
Food allergies	The number of CD19+CD5+ regulatory B cells is reduced in patients with milk allergy compared to the control group	Noh et al., 2010 [98]
	CD19+CD25+CD71+CD73- regulatory B cells have reduced ability to secrete IL-10. Proliferation of CD4+CD25+ regulatory B cells is increased in patients with ulcerative colitis	Sun et al., 2019 [102]
	The number of TGF-β+CD19+CD5+ and CD19+CD5+Foxp3+ regulatory B cells is reduced in patients with cow’s milk allergy	Sun et al., 2019, Sampath 2020 [102,110]
	CD19+CD25+CD71+ regulatory B cells secrete reduced amounts of IL-10	Kaplan 2015 [101]
Allergen immunotherapy	Increased number of IL-0+ and/or IL-1RA+ regulatory B cells in patients using AIT with house dust mite allergens	Boonpiyathad et al., 2019 [105]
	Allergen immunotherapy induces the development of IL-35+ and IL-10+ regulatory T lymphocytes and regulatory B lymphocytes	Sharif 2019 [106]
	During the allergy season, the number of IL-10 regulatory B cells is increased in people who are allergic to a given alergen	Shamji et al., 2019 [95]
	In beekeepers and patients after AIT, an increase in the number of D73-CD25+CD71+IL-10+ BR1 lymphocytes is observed	Boonpiyathad et al., 2017 [108]
	The percentage of IL-10 regulatory B cells specific for bee venom allergens is increased compared to nonspecific B cells. Naïve CD27- regulatory B cells are characterized by increased selective IgG4 production compared to IL-10-naïve cells	Van de Veen et al., 2013 [49]

## Data Availability

Not applicable.
